# A novel biomarker assay for cancer immunotherapy development based on myeloid derived supressor cells (MDSCs)

**DOI:** 10.1186/2051-1426-1-S1-P103

**Published:** 2013-11-07

**Authors:** Henry Hepburne-Scott

**Affiliations:** 1 Serametrix Corporation, Carlsbad, CA, USA

## 

It has long been known that myeloid derived suppressor cells (MDSCs) inhibit the anti-tumor immune response. And recently a relationship between MDSC levels and patient outcomes to cancer immunotherapy was discovered. Studies conducted at the Memorial Sloan Kettering Cancer Center in New York showed that patients with reduced levels of circulating MDSCs were more likely to respond favorably to Ipilimumab, an immunotherapy approved for the treatment of melanoma. This raises the exciting prospect that baseline levels of MDSCs may help drug developers and physicians identify or select those patients who are most likely to benefit from immune-based cancer treatments. However the development of a robust and commercially available MDSC biomarker assay presents many challenges, from sample handling and logistics to an objective and reproducible definition of this elusive cell type. Here we present data from our MDSC assay development program and discuss the merits of applying this assay to biomarker studies as part of the clinical development of novel cancer therapies.

